# Targeting skeletal muscle health with exercise in people with type 1 diabetes: A protocol for HOMET1D, a prospective observational trial with matched controls

**DOI:** 10.1371/journal.pone.0303448

**Published:** 2024-05-22

**Authors:** Irena A. Rebalka, Kenneth S. Noguchi, Kayla R. Bulyovsky, Matthew I. Badour, Emma S. Juracic, Khandra Barrett, Aditya Brahmbhatt, Baraa Al-Khazraji, Zubin Punthakee, Christopher G. R. Perry, Dinesh A. Kumbhare, Maureen J. MacDonald, Thomas J. Hawke

**Affiliations:** 1 Department of Pathology & Molecular Medicine, McMaster University, Hamilton, Ontario, Canada; 2 School of Rehabilitation Science, McMaster University, Hamilton, Ontario, Canada; 3 Department of Kinesiology, McMaster University, Hamilton, Ontario, Canada; 4 School of Kinesiology & Health Science, Muscle Health Research Centre, York University, Toronto, Ontario, Canada; 5 Department of Medicine, McMaster University, Hamilton, Ontario, Canada; 6 Department of Medicine, University of Toronto, Toronto, Ontario, Canada; PLOS: Public Library of Science, UNITED KINGDOM

## Abstract

**Introduction:**

Individuals with type 1 diabetes (T1D) experience a complex set of alterations to skeletal muscle metabolic, neuromuscular, and vascular health; collectively referred to as diabetic myopathy. While the full scope of diabetic myopathy is still being elucidated, evidence suggests that even when individuals with T1D are physically active, indices of myopathy still exist. As such, there is a question if adherence to current physical activity guidelines elicits improvements in skeletal muscle health indices similarly between individuals with and without T1D. The objectives of this trial are to: 1) compare baseline differences in skeletal muscle health between adults with and without T1D, 2) examine the association between participation in a home-based exercise program, detraining, and retraining, with changes in skeletal muscle health, and 3) examine the roles of age and sex on these associations.

**Methods and analysis:**

This will be a prospective interventional trial. Younger (18–30 years) and older (45–65 years) males and females with T1D and matched individuals without T1D will engage in a four-phase, 18-week study sequentially consisting of a one-week lead-in period, 12-week exercise training program, one-week detraining period, and four-week retraining period. The exercise program will consist of aerobic and resistance exercise based on current guidelines set by Diabetes Canada. Metabolic, neuromuscular, and vascular outcome measures will be assessed four times: at baseline, post-exercise program, post-detraining, and post-retraining. Differences in baseline metrics between those with and without T1D will be examined with independent sample t-tests, and with two-way analyses of variance for age- and sex-stratified analyses. Changes across the duration of the study will be examined using mixed-model analyses.

**Dissemination:**

Findings from this research will be shared locally and internationally with research participants, clinicians, diabetes educators, and patient advocacy organizations via in-person presentations, social media, and scientific fora.

**Trial registration number:**

NCT05740514.

## Introduction

More than 8.4 million people worldwide are currently impacted by type 1 diabetes mellitus (T1D), resultant from autoimmune destruction of the insulin-producing pancreatic beta cells [1]. Despite rigorous and continuous exogenous insulin therapy, those with T1D can develop severe complications, such as cardiovascular, retinal, and renal disease, that impact quality of life and lifespan; with mortality occurring more than a decade earlier than those without T1D [2–4].

Another complication that develops in those with T1D is diabetic myopathy; alterations to skeletal muscle health [5–10]. Notable when considering activities of daily living, individuals with T1D display decreased skeletal muscle strength and an increased presence of sarcopenia when compared to their matched controls [11].

Contractility, morphology, metabolism, and the coordination of myocytes with their surrounding microvascular and neuromuscular network are key characteristics of skeletal muscle, and the roles of skeletal muscle health, collectively encompassing its metabolic, microvascular, and neuromuscular components, are well-established for the general population. The maintenance of skeletal muscle function via exercise creates downstream effects on overall health, improving blood glucose management, insulin sensitivity, cardiovascular outcomes (blood pressure regulation and oxygen transport), and the capacity to undertake activities of daily living [12–14]. However, the impact of T1D on all facets of collective skeletal muscle health, as well as the comparative effects of exercise training in those with and without T1D is not as well established.

While current exercise guidelines for individuals with diabetes recommend a minimum of 150 minutes of moderate-to-vigorous aerobic exercise and two sessions of resistance exercise each week [15], these guidelines were mostly generalised from studies conducted in individuals with other metabolic conditions such as obesity, pre-diabetes, and type 2 diabetes [15]. It is currently unknown whether these recommendations are specifically appropriate for people with T1D, as, to date, no study has examined whether adherence to current recommendations are sufficient to improve systemic or musculoskeletal health variables in persons with T1D. In fact, recent studies suggest that recreational physical activity is insufficient in preventing skeletal muscle metabolic deficiencies in young and older adults with uncomplicated T1D [9], and that individuals with T1D achieve blunted exercise adaptations following exercise training compared to those without T1D [16]. Furthermore, considering the sexual dimorphism observed in skeletal muscle metabolism and function in people with T1D [6, 8, 10], as well as the known interactions between sex and age in people with and without T1D [6], it is also unclear whether the effects of exercise are different between these subgroups.

With the absolute need for a greater understanding of the metabolic, microvascular, and neuromuscular components of skeletal muscle in T1D as the foundation, the objectives of this trial are three-fold: 1) compare baseline differences in skeletal muscle health between individuals with and without T1D, 2) examine the association between participation in a home-based exercise training program, detraining, and retraining with changes in skeletal muscle health, and 3) examine the roles of age and sex on skeletal muscle health at baseline, and on longitudinal changes following this program.

## Methods

The following protocol was described in accordance with the Standard Protocol Items: Recommendations for Interventional Trials (SPIRIT) guidelines and was prospectively registered on ClinicalTrials.gov (NCT05740514).

### Trial design and setting

This study is a non-randomized, prospective controlled interventional trial taking place in Ontario, Canada. The trial protocol will span four distinct phases over 18 weeks: a lead-in period (one week), a home-based exercise training program (12 weeks), a detraining period (one week), and a retraining period (four weeks). All measures will be evaluated over five visits, at 0, 1, 13, 14, and 18 weeks. The exercise training intervention will be conducted at home.

### Eligibility criteria

Males and females with and without T1D will be eligible if they are younger (18 to 30 years) or older (45 to 65 years) adults and are sedentary or recreationally active (defined by self-report of <150 minutes of moderate to vigorous physical activity [MVPA] per week). Participants will complete a standardized medical questionnaire, Get Active Questionnaire (Canadian Society for Exercise Physiology (CSEP); Ottawa, ON, Canada), and global physical activity questionnaire (GPAQ) [17] to confirm their eligibility and suitability for the study. Participants with T1D must have a confirmed diagnosis or be willing to have their status confirmed using a C-peptide or anti-glutamic acid decarboxylase test. Table 1 contains the full eligibility criteria, which are the same for those with and without T1D. Participants with and without T1D will be matched 1:1 by age (±4 years), sex, BMI (±4 kg/m^2^), and by statin and blood pressure medication use (if relevant).

**Table 1:** Participant eligibility criteria.

Inclusion	Exclusion
Sedentary or recreationally active (<150 mins moderate-to-vigorous intensity physical activity per week) Aged 18–30 (*young adult cohort)* or 45–65 *(older adult cohort)* years old at the time of study start Confirmed diagnosis of T1D *(T1D cohort only)*	Chronic use of anti-inflammatory, glucocorticoid, or other pain-relief medication History of daily cannabis, tobacco, or nicotine use in the previous six months Other health conditions precluding exercise participation Type 2 diabetes or prediabetes Atypical or Grade 2b diabetic sensorimotor polyneuropathy >1 lifetime event of hospitalization for diabetic ketoacidosis

T1D = Type 1 diabetes

### Physical activity and exercise intervention

**Fig 1:**
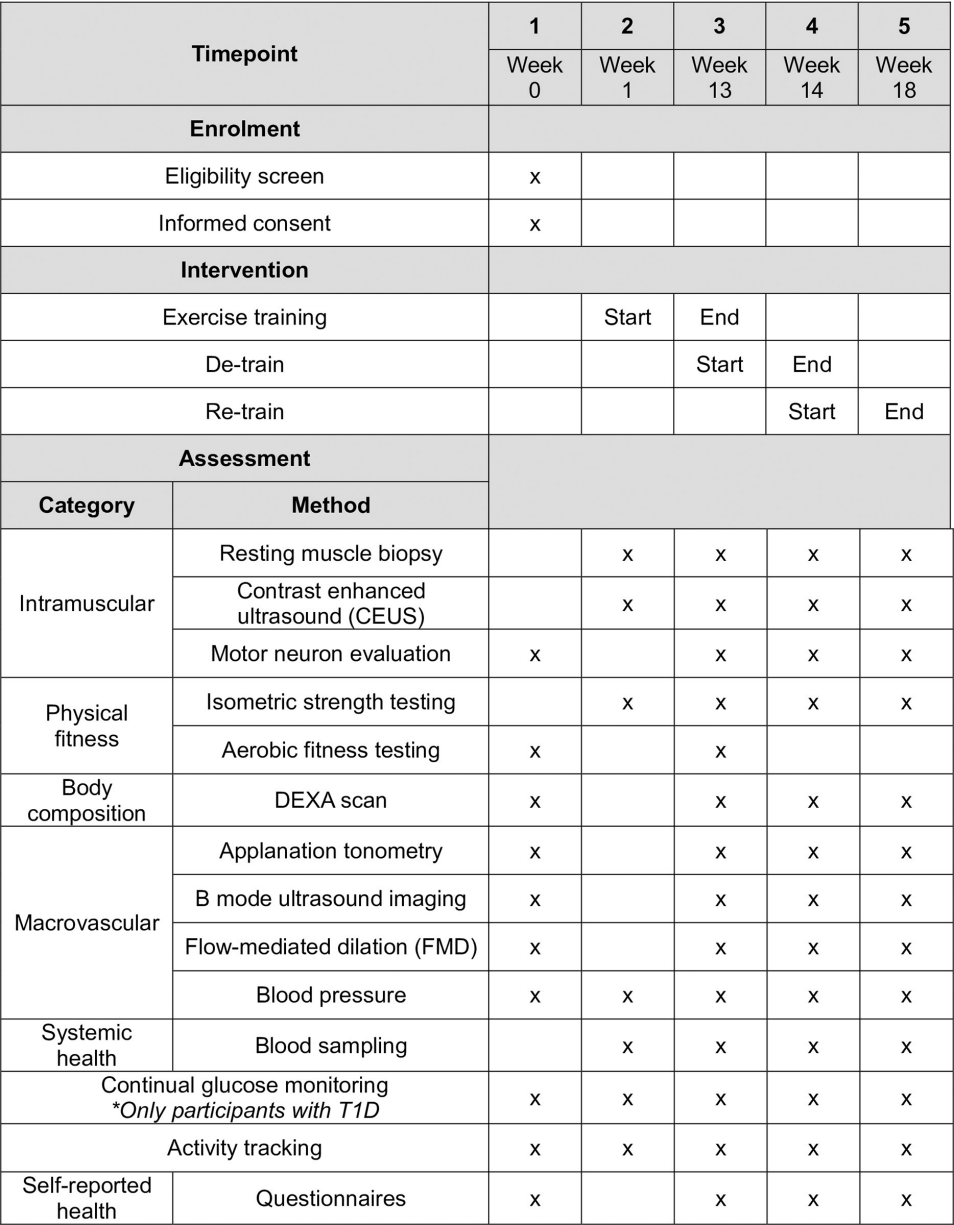
HOMET1D trial timeline and outcome measures assessed.

Participants will undergo home-based exercise training up to five days per week for 12 weeks during the exercise training program phase, and again for four weeks during the retraining phase. See Fig 1 for a representation of this study timeline. This program is modeled from the current Diabetes Canada exercise guidelines [15] and the 24-hour movement guidelines from CSEP [18].

During the first two weeks of the exercise training program, participants will engage in introductory movements and brisk walks to improve confidence and program retention. Thereafter, participants will be instructed to engage in at least 150 minutes of MVPA, defined as above 64% of peak heart rate (HR_peak_), derived from a published equation for maximal heart rate (i.e., HR_peak_ = 208 –[0.7*age]) [19]. Participants and the research team will track participant activity levels and heart rate using a commercially available activity monitor (Garmin Venu Sq; Garmin; Olathe, KS, USA). Modalities to achieve target MVPA include, but are not limited to, brisk walking, bicycling, jogging, dancing, jumping rope, swimming, or high-intensity interval training, in bouts of ten minutes or more. Participants will also be asked to engage in a minimum of two days of resistance exercise per week, defined as brief repetitive exercises with weights, weight machines, resistance bands, or body weight. To facilitate achievement of target MVPA and resistance training, participants will also be provided with an exercise calendar, an exercise guide containing suggestions for different modalities and movements, and a set of resistance bands as required.

The research team will communicate activity level summaries with the participants via email on a weekly basis during the exercise training phase and the retraining phase to reinforce adherence. If a study participant is not compliant with the target MVPA and resistance exercise for two consecutive weeks, they will be withdrawn from study participation.

Individuals with T1D will also be supplied with a Dexcom continuous glucose monitoring (CGM) system (Dexcom; San Diego, CA, USA) to support safety during exercise (e.g., reduce risk of hypoglycemia) and to permit assessment of clinically relevant CGM-related metrics (e.g., time-in-range, glycemic variability, etc.) for the entire duration of this study.

### Detraining period

After the 12-week exercise training program, all participants will undergo a seven-day ‘detraining’ period involving unilateral lower-limb immobilization of the left leg [20]. A hinge-jointed immobilization brace (DonJoy X-Act ROM; DJO Global; Lewisville, TX, USA) will be applied and locked to 60° of knee flexion to prevent normal weight-bearing while allowing knee extensors to remain relaxed. Participants will be given crutches and trained on their use to aid in ambulation. Participants will be permitted to remove the brace for sleep and will perform daily range of motion movements at the knee and ankle to mitigate the risk of vascular and musculoskeletal complications.

### Outcomes

Participants will enter the laboratory for data collection visits after a 24-hour abstinence from alcohol, caffeine, and exercise. A detailed description of outcomes and their collection and mesurement procedures are provided in Table 2.

**Table 2:** Key outcome measures and protocol details.

Type of measure	Variables	Protocol details
Intramuscular	Mitochondrial density and bioenergetic measures including mitochondrial respiration (pyruvate-malate supported, palmitoyl-CoA supported, palmitoylcarnitine supported) H_2_O_2_ emissions, and calcium retention capacity [6, 8–10]. Muscle satellite cell content; indicies of muscle damage [21]; microvasculature [6]; ultrastructural morphometrics [9]	A resting Bergstrom skeletal muscle biopsy will be conducted. Briefly, an area superficial to the vastus lateralis muscle of the thigh will be cleaned and local lidocaine anesthetic will be injected. A small incision will be made in the skin via scalpel blade, into which the biopsy needle will be inserted. A small piece of muscle (~100 mg) will be removed by the biopsy needle. The incision will then be closed and bandaged. Techniques such as mitochondrial bioenergetics (Oroboros Instruments O2k modular system for high-resolution respirometry), histology, immunoblotting/fluorescent measures, and electron microscopy will be used to evaluate all indicies of interest.
	Microvascular function and perfusion: flow velocity; blood volume; blood flow (all AU); acoustic intensity (AU)	Microvascular function and perfusion will be evaluated within the vastus lateralis using Contrast Enhanced UltraSound (CEUS). Lipid encapsulated sulfur-hexafluoride filled microspheres will be released into systemic circulation via intravenous infusion through a forearm vein to steady state to enhance ultrasound images. Once steady state is achieved, a brief, high mechanical index ultrasound pulse is initiated to destroy microspheres in a region of interest and their replenishment is recorded in a cineloop. Ultrasound cineloops will be taken at rest and following a submaximal bout of resistance exercise.
	Motor neuron evaluation: Voltage (mV); discharge rate (pps); mean firing rate (pps); conduction velocity (m/s)	Non-invasive high-density surface electromyography (EMG; Sessantaquattro 64-channel EMG) signals of the vastus lateralis will be recorded with a two-dimensional adhesive electrode grid whose cavities will be filled with conductive paste once affixed to the skin. The EMG signals will be recorded in a mono-polar mode and converted to digital data through a multi-channel amplifier for evaluation and analysis.
Physical fitness	Quadricep maximal isometric strength (N)	Participants will maximally push against a padded lever set to a knee joint angle of 90 degrees for a five-second period. This will be repeated three times with 30 seconds rest between repetitions. The maximum value will be reported.
	Isometric grip strength (kg)	Participants will perform two repetitions of maximal squeezing with each hand on a handgrip dynamometer, and if the two measurements differ by 5%, a third measurement will be performed. The maximum value will be reported.
	Aerobic fitness (mL/kg/min); peak power (W)	For individuals who answered “No” to all questions of the CSEP Get Active Questionnaire, a VO_2_Peak test will be conducted. On a cycle ergometer, participants will perform a two-minute warm-up followed by cycling at 70–90 RPM under increasing resistance (one Watt each two seconds). Testing will stop when volitional exhaustion is reached (i.e., falling below a cadence of 60 revolutions per minute for > one second). Participants will wear a face mask connected to the metabolic cart for real-time assessment of oxygen uptake and carbon dioxide expenditure. For individuals who answered “Yes” to any questions within the CSEP Get Active Questionnaire, a PWC130 test, consisting of two consecutive six-minute bouts on a cycle ergometer will be completed. The selected resistance will produce a heart rate of approximately 100-110bpm in the first bout, and approximately 130-140bpm in the second bout.
Body composition	Total and compartmental body mass index (kg/m^2^); fat and fat-free mass (kg, %)	A dual-energy X-ray absorptiometry (DEXA) scan will be taken to gain a comprehensive overview of body composition. Participants will lay supine for seven minutes for each whole-body scan.
Macrovascular	Arterial stiffness (m/s)	Arterial stiffness (pulse wave velocity (PWV)) will be measured via applanation tonometry. Two tonometers will be placed superficially over the arterial regions of interest (carotid-femoral and femoral-foot) for 20 heart cycles to calculate central and peripheral arterial stiffness, respectively, using the formula: PWV = distance / transit time. PWV will be assessed at rest and at various timepoints following a bout of maximal aerobic exercise.
	Carotid intima-media thickness (mm)	B mode ultrasound will be used to capture images of the common carotid artery which will be analyzed using semi-automated edge tracking software to determine carotid intima-media thickness.
	Vasodilatory function (%)	Endothelial-dependent vasodilatory function will be assessed using brachial artery flow-mediated dilation (FMD). A blood pressure cuff will be placed around the forearm distal to the elbow, be inflated above systolic blood pressure to ~200 mmHg, and held at this pressure for five minutes. A Doppler ultrasound probe will be placed on the upper arm (below the biceps) and moved until the best signal is found. Images of brachial artery diameter and blood flow velocity will be taken at rest (before cuff inflation), prior to cuff deflation (end of five-minute ischemic period), and following cuff deflation for three minutes. FMD will be calculated using this formula: FMD% = ((peak diameter—baseline diameter) / baseline diameter) × 100%.
	Carotid distensibility (mmHg)	This assessment involves the simultaneous measurement of B mode ultrasound of the right carotid artery and applanation tonometry of the left carotid artery for 10 heart cycles.
	Blood pressure (mmHg); heart rate (bpm)	Following ten minutes of seated rest, systolic and diastolic blood pressure (mmHg) will be collected using an automated oscillometric blood pressure monitor. The average of three trials will be taken, with at least one minute rest between measurements. During supine macrovascular assessments (FMD, PWV, cIMT), blood pressure and heart rate will be continuously monitored via Finometer and 3-lead ECG, respectively.
Systemic health	Blood markers, including: glucose; insulin; free fatty acids; cholesterol; triglycerides; HDL/LDL; HbA1c; lactate; creatinine; urea; uric acid; potassium; vascular health biomarkers; tryptophan metabolites	Blood samples (~20mL) will be drawn from a vein on the anterior compartment of the forearm. Serum and plasma samples will be isolated using standardized protocols, and appropriate analysis (core facility analysis, ELISA kit, etc.) will be conducted to quantify each factor of interest.
CGM Data (only participants with T1D)	Time in, below, above range (%); average blood glucose (mM); mean amplitude of glycemic excursion (mM)	Participants with T1D will wear a Dexcom CGM system for the 18-week study duration. Participants will be instructed on how to insert, remove, and monitor the Dexcom device and platform. Participants and investigators will actively monitor blood glucose to help facilitate safe exercise during the study.
Garmin Activity Tracker Data	Heart Rate (bpm); blood oxygen saturation (%); physical activity duration (mins) and intensity (% max hr); sleep duration (mins); respiration rate (breaths/min)	Participants will wear the Garmin Venu Sq watch during the day and night for the 18-week duration of the study. Participants will be trained on its use, including how to activate “*activity mode*” to record daily exercise throughout the study. These metrics will be accessible in real time by the research team to track study and exercise progress, as well as collect data.
Self-reported health	DASS-21	The DASS-21 is used in clinical and non-clinical settings to measure depression, anxiety, and stress [22]. It has 21-items and is rated on a four-point scale (0 = “did not apply to me at all” to 3 = “applied to me very much or most of the time”).
	DIDP (only participants with T1D)	The DIDP is used to quantify health-related quality of life in individuals with diabetes [23]. It is a six-item questionnaire that asks about six dimensions of life: physical health, financial situation, relationships, leisure activities, work or studies, and emotional wellbeing. Each item is rated on a seven-point Likert scale (1 = very positive impact, 7 = very negative impact) and is scored both separately and together by summing all responses.

The co-primary outcome measures (mitochondrial density and muscle satellite cell content) will be assessed from a resting skeletal muscle biopsy of the vastus lateralis muscle.

Secondary outcomes include measures of physical fitness such as muscle strength and aerobic fitness. Quadriceps maximal isometric strength (Newtons [N]) will be assessed using an isokinetic dynamometer (System 3; Biodex Medical Systems; Shirley, NY, USA). Isometric grip strength (kilograms [kg]) will be measured bilaterally using a hand grip dynamometer (Jamar Hydraulic Hand Dynamometer 5030J1; Sammons Preston Inc.; Bolingbrook, IL, USA). Aerobic fitness (mL/kg/min) will be evaluated on a cycle ergometer (Excalibur Sport; Lode BV; Groningen, The Netherlands).

Tertiary outcomes include assessments of body composition, macrovascular, microvascular, and systemic health indicators, as well as interrogation of continuous glucose monitoring and activity tracker data. Body composition will be assessed using dual-energy X-ray absorptiometry scan (Lunar iDXA; GE HealthCare; Chicago, IL, USA). Macrovascular assessments include arterial stiffness (measured within carotid-femoral and femoral-foot regions via applanation tonometry), carotid intima-media thickness (determined from images acquired via B mode ultrasound), endothelial-dependent vasodilatory function of the brachial artery, carotid distensibility, resting heart rate, as well as resting systolic and diastolic blood pressures. Microvascular function and perfusion will be assessed using contrast enhanced ultrasound (CEUS) of the vastus lateralis [24]. Systemic health markers drawn from blood samples will include analysis of several factors including glucose, insulin, free fatty acids, cholesterol, triglycerides, high-density lipoprotein (HDL), low-density lipoprotein (LDL), glycated hemoglobin (HbA1c), lactate, creatinine, urea, uric acid, potassium, plasminogen activator inhibitor 1 (PAI-1), and tryptophan metabolites.

Quaternary outcomes collected via surface electromyography include intramuscular measures such as motor unit recruitment, recruitment threshold, discharge rate, and conduction velocity. Other intramuscular outcomes derived from a skeletal muscle biopsy of the vastus lateralis muscle include, but are not limited to, mitochondrial bioenergetic measures, and analyses of key metabolic and morphological indices (using histological and immuno-blotting/fluorescent assays and electron microscopy).

Throughout this study, emotional state will be evaluated in all study participants using the Depression, Anxiety and Stress Scale (DASS-21) [22]. Health-related quality of life will be evaluated in participants with T1D using the DAWN Impact of Diabetes Profile (DIDP) [23].

### Blinding

As all participants will partake in exercise training and detraining, neither participants nor investigators will be blind to these interventions. Moreover, for safety, outcome assessors will not be blind to T1D diagnosis during visits. However, investigators conducting tissue analyses (primary, partial tertiary, and partial quaternary outcomes) and statistical analyses (all outcomes) will be blind to T1D diagnosis, age, and sex. A password-protected study key with de-identified participant identification numbers will be used for blind analyses. T1D diagnosis, age, and sex will be revealed once analyses are complete.

### Sample size and recruitment

Sample size calculations were performed for the primary objective of comparing sex- and age-stratified baseline differences in the primary outcomes of interest, namely mitochondrial density and muscle satellite cell content. Based on our previous work, older adults with T1D versus without T1D have an expected difference in mitochondrial density of 2.003% (density = mean mitochondrial size *×* number of mitochondrial fragments per tissue area, expressed as percent area of muscle) and a pooled standard deviation of 1.395%. For an independent-sample t-test, 17 older adults per sex and group (n = 68) would be sufficient to detect a difference with 80% power at an alpha level of 0.05. Younger adults with and without T1D have an expected difference in satellite cell content of approximately 3.43 Pax7-positive satellite cell nuclei per 100 myofibers and a pooled standard deviation of 3.08. Thus, 13 younger adults per sex and group (n = 52 total) would be sufficient to detect a difference in satellite cell content. Both values were increased to a final sample size of 96 older adults and 72 younger adults, providing a 40% excess to account for attrition.

Recruitment began on October 1, 2023. Participants are currently being recruited from within the greater Hamilton, Niagara, and Toronto areas via printed posters and online platforms. For in-person advertisement, posters are placed within classified advertisements, around the McMaster University campus, and within local cafés, fitness centres, pharmacies, and stores. Advertisements are also posted online via social media platforms (e.g., Facebook, X) and T1D-centered platforms (e.g., ConnectedinMotion.ca, ConnecT1D.ca, and BeyondType1.org).

### Statistical analysis

Continuous data will be described using means and standard deviations or medians and interquartile ranges for normally and non-normally distributed variables, respectively. Categorical data will be presented using frequencies and percentages.

Primary analyses will determine baseline differences in skeletal muscle health between those with and without T1D. This will be conducted for the full sample and separately for age and sex subgroups. To compare differences in the primary outcomes between people with and without T1D, we will use an independent sample t-test (if normally distributed) or Mann-Whitney U test (otherwise). The same analyses will be repeated for all continuous secondary to quaternary outcomes. The independent variable of interest in each t-test will be T1D diagnosis (two levels: with vs without T1D). Additional exploratory analyses will examine the impact of age, or sex, and T1D diagnosis using two-way analyses of variance with an interaction between T1D diagnosis with age (two levels: younger vs older adults) or sex (two levels: male vs female).

Secondary analyses will examine longitudinal changes associated with exercise training, detraining, and retraining. Mixed model analyses will be performed for each outcome measure. Timepoint (four levels: baseline (week 0,1), post-training (week 13), post-detraining (week 14), post-retraining (week 18)) and T1D diagnosis will be the primary independent variables of interest. To examine longitudinal changes between those with and without T1D, interaction terms between timepoint and T1D diagnosis will be included in each model. To examine the role of age and sex in these models, a three-way interaction between timepoint, T1D diagnosis, and either age or sex will be included. Random intercepts and slopes will be tested in each model. Independent and unstructured covariance structures will be explored. The Bayesian Information Criteria (BIC) and log likelihood ratio (LR) test will be used to determine the best fitting model. Lower BIC values and significant LR test indicates superior model fit.

If the interaction is significant in either primary or secondary analyses, Sidak-adjusted pairwise comparisons will be conducted. Irrespective of significance in the interactions, the lower-order independent variables will be retained. Significant outliers will be considered for removal from analysis, but data from the full and modified models will be presented. Data points will be considered outliers if they are: theoretically implausible, contribute to a >10% difference in beta-coefficients and/or change the interpretation of the model. Missing data for primary analyses will be handled using multiple imputation if it is considered missing at random. All analyses and data visualization will be performed on Stata SE (version 17.0; College Station, TX, USA), GraphPad Prism (GraphPad Software; Boston, MA, USA), or R (The R Foundation for Statistical Computing; Vienna, Austria). The accepted significance level will be set to *p*<0.05.

## Ethics and dissemination

This study protocol has received full and final ethics approval by the Hamilton Integrated Research Ethics Board (HiREB #15516). All protocol modifications, if any, will be approved by the HiREB, and participants may be asked to re-consent to the study if deemed necessary by the ethics board.

### Consent process

Once the details of the study have been discussed with a potential participant and all inclusion criteria have been confirmed, participants will be asked to read the informed consent form. After questions and concerns have been addressed, potential participants will be asked if they would like to participate in the study, and consent forms will be signed. The consent process will be done at McMaster University, and participants will be provided the contact information for the research team. Given that this study takes place over five visits, participants will be closely monitored throughout the duration of the study and will have several opportunities to ask questions or identify concerns. If a participant wishes to withdraw from the study, they will be free to do so at any time by contacting the research team. Any withdrawals will be documented, and data prior to withdrawal will be kept within the study. Participants will be compensated for their time commitment up to the time of their withdrawal.

### Data privacy, safety, and supervision

All identifying information will be replaced by a code to link data collected with each participant. All data collected during this study will remain confidential and stored in locked offices and on password protected computers to which only the investigators have access. Identifying information such as names and contact information will be held by TJH in a locked filing cabinet within a locked office. All tissue and blood sample collection tubes will be labeled with each participant’s de-identified study ID to ensure that confidentiality is maintained throughout the course of the study.

With participant consent, muscle and blood samples collected for this study may be stored so that new factors involved in skeletal muscle health can be tested as they become available. Participants will be provided the following three options at the time of informed consent: samples not stored for future testing, samples stored for future testing without identifying data linking participant and samples, and samples stored for future testing with identifying data linking participant and samples.

Participation will be fully supervised by an investigator in order to ensure safety and compliance. All invasive procedures will be performed by trained personnel under the care of a physician (DAK). Participants have been instructed to contact the research team via phone or email should any questions or concerns arise during their study participation.

### Patient and public involvement, and knowledge dissemination

Individuals with T1D and endocrinologists were involved in setting the current research questions and outcome measures, were consulted in the visit day implementation of the current research design, and have played a large role in study recruitment.

The complete findings of this study will be shared with research participants in a newsletter-style report suitable for a non-specialist audience, and will also be published by the research team in peer-reviewed journals, and presented at regional, national, and international conferences. Addditionally, the results of this study will be disseminated to clinicians, diabetes educators, and agreeable patient advocacy organizations in a manner that will be facilitated by the preference of our participants. Our target clinicians include endocrinologists, and clinicians partnered with specialist care providers such as LMC Healthcare (www.lmc.ca). Target patient advocacy organizations may include Connected in Motion (ConnectedinMotion.ca; a Canadian charitable organization that provides peer-based experiential diabetes education and fosters sport and outdoor adventure pursuits), ConnecT1D (ConnecT1D.ca; a Canadian organization which connects community members with T1D to the latest research findings about T1D treatment and management), Beyond Type 1 (BeyondType1.org; international non-profit organization with a focus on T1D education, advocacy, and research), and The Juveline Diabetes Research Foundation–Canada (JDRF.ca; a T1D advocacy group that facilitates community engagement and supports scientific research aimed at curing, treating, and preventing T1D and its complications).

## Conclusion

The present trial will provide novel and comprehensive insight into the impact of T1D on human skeletal muscle health, as well as systemic health and physical fitness, across the lifespan and between sexes. More specifically, these results will determine whether the current exercise guidelines for those with T1D are sufficient to maintain skeletal muscle health, and determine the impact of pauses in exercise programs, such as that seen during illness or injury. Ultimately, this research will provide the mechanistic insight necessary for the development of appropriate exercise prescriptions to maximize skeletal muscle health and improve the lives of those with T1D.

## Supporting information

S1 Checklist (DOCX)

S1 Protocol (DOCX)
