# Supplementary material for: Targeting skeletal muscle health with exercise in people with type 1 diabetes: A protocol for HOMET1D, a prospective observational trial with matched controls
Source: PLoS One. 2024 May 22;19(5):e0303448. doi: 10.1371/journal.pone.0303448 (PMC11111001; doi:10.1371/journal.pone.0303448)
Supplement: S1 Protocol — (DOCX) [file pone.0303448.s002.docx]

**HOMET1D STUDY PROTOCOL**

**STUDY TITLE:**

**Healthy Outcomes for Muscle with Exercise in T1D (HOMET1D)**

**FUNDING SOURCE:**

Canadian Institutes of Health Research (CIHR) Project 180340

Targeting vascular and skeletal muscle health to improve quality of life in males and females with Type 1 Diabetes

**TABLE OF CONTENTS**

[1. STUDY BACKGROUND AND OBJECTIVES 2](#_Toc132531731)

[1.1 BACKGROUND 2](#_Toc132531732)

[1.2 OBJECTIVES 2](#_Toc132531733)

[2. STUDY POPULATION, INCLUSION, AND EXCLUSION CRITERIA 3](#_Toc132531734)

[2.1 STUDY POPULATION 3](#_Toc132531735)

[2.2 EXCLUSION CRITERIA 3](#_Toc132531736)

[2.3 SAMPLE SIZE 4](#_Toc132531737)

[3. STUDY DESIGN AND MEASUREMENT INSTRUMENTS 4](#_Toc132531738)

[3.1 STUDY SUMMARY 4](#_Toc132531739)

[3.2 DETAILS OF INITIAL STUDY DOCUMENTATION 5](#_Toc132531740)

[3.3 DETAILS OF STUDY VISITS 6](#_Toc132531741)

[3.4 DETAILS OF EXERCISE TRAINING 10](#_Toc132531742)

[3.5 DETAILS OF DETRAINING PERIOD 11](#_Toc132531743)

[3.5 DETAILS OF RE-TRAINING PERIOD 11](#_Toc132531744)

[4. DATA ANALYSIS 12](#_Toc132531745)

[5. BUDGET OVERVIEW 12](#_Toc132531746)

[6. SUBJECT RECRUITMENT 13](#_Toc132531747)

[6.1 OVERVIEW 13](#_Toc132531748)

[6.2 RECRUITMENT POSTERS 14](#_Toc132531749)

[6.3 SOCIAL MEDIA, ONLINE, CLASSIFIED AD RECRUITMENT 14](#_Toc132531750)

[7. LITERATURE CITED 15](#_Toc132531751)

# 1. STUDY BACKGROUND AND OBJECTIVES

## 1.1 BACKGROUND

Type 1 diabetes mellitus (T1D) results from autoimmune destruction of the insulin-producing pancreatic beta cells. Despite exogenous insulin therapy via daily insulin injections or insulin pump usage, individuals with T1D develop severe complications such as cardiovascular and renal disease, largely due to recurrent excessive fluctuations in blood glucose levels, referred to as dysglycemia,^1^ and the subsequent development of insulin resistance.^2–17^ These complications impact the quality of life and lifespan of those with T1D; estimated at fifteen years less than those without T1D.^18^

Contractility, morphology, metabolism, and the coordination of myocytes (muscle cells) with their surrounding microvascular and neuromuscular network are key characteristics of skeletal muscle, the organ system primarily responsible for our physical and metabolic capacities. The maintenance of this multitude of components and characteristics (collectively referred to as skeletal muscle health) is important for our overall well-being, with improvements in skeletal muscle health affecting blood glucose management, insulin sensitivity, cardiovascular outcomes (blood pressure regulation and oxygen transport), and the capacity to undertake activities of daily living.^19–25^ **While these statements are well-established for healthy individuals, our understanding of the impact of T1D on collective skeletal muscle health is limited, and the benefits of exercise training on T1D skeletal muscle health are virtually unknown.**

New data published by our lab highlights impairments in skeletal muscle metabolism, microvasculature, and function with T1D.^26–29^ Preliminary findings also suggest that a higher intensity or volume of exercise prescription may be warranted in those with T1D to maintain or improve muscle health relative to matched non-T1D counterparts. This is concerning, however, as current exercise recommendations for those with T1D are the same as for those without T1D.

## 1.2 OBJECTIVES

To come to a thorough understanding of the integrated impairments to skeletal muscle health and its coordinated response to exercise training in T1D, we will investigate the following specific three objectives. A brief methodological overview is provided with each objective, and methodological details may be found in Section 3 – Study Design and Measurement Instruments, below.

**Objective 1: Define the impact of T1D on systemic and muscle health**.

During a baseline period, study participants will undergo strength/aerobic fitness testing, body composition, blood analysis, glucose/activity/heart rate monitoring, muscle biopsy, neuromuscular assessments, and assessments of macrovascular and microvascular structure and function.

*Metric results acquired from visits 1/2 (see Section 3.1 Study Summary, below), will be compared between individuals with T1D and matched controls.*

**Objective 2: Identify the effects of exercise on systemic and skeletal muscle health in T1D.**

Following Objective 1, subjects will undergo a combined aerobic and resistance training program for twelve weeks. All tests from Objective 1 will be repeated.

*Metric results acquired from visits 1/2 (see Section 3.1 Study Summary, below), will be compared to metric results acquired from visit 3 for all individuals.*

**Objective 3: Determine the impact of detraining on systemic and muscle health in T1D.**

Following Objective 2, all subjects will undergo a detraining period consisting of unilateral knee-bracing for seven days, after which all tests from Objective 1 will be repeated. Subjects will then resume the combined aerobic and resistance training program for four weeks, after which all tests from Objective 1 will again be repeated.

*Metric results acquired from visit 3 (see Section 3.1 Study Summary, below), will be compared to metric results acquired from visit 4 for all individuals. Similarly, metric results acquired from visits 3 and 4 will also be compared to metric results acquired from visit 5 for all individuals.*

# 2. STUDY POPULATION, INCLUSION, AND EXCLUSION CRITERIA

## 2.1 STUDY POPULATION

Young (**18 to 30 years**) and older (**45 to 65 years**) adult females and males with and without confirmed T1D will be recruited to participate in this proposed investigation. All adults with T1D and without T1D will be matched for age, sex, and BMI at study onset. Subjects will also be matched for medication use, particularly statin and blood pressure medications. Participants must complete a standard medical questionnaire and physical activity readiness questionnaire (PARQ+) at study onset. Subjects should be sedentary or recreationally active, as defined by self-reported activity levels below the recommended 150-minute minimum of moderate-to-vigorous intensity physical activity per week, as recommended by Health Canada and Diabetes Canada.

## 2.2 EXCLUSION CRITERIA

Subjects will not be eligible to participate in or will be released from this study if they match any one of the following criteria:

- Chronic use of anti-inflammatory, glucocorticoid, or other pain-relief medication
- History of daily cannabis, tobacco, or nicotine use within six months of study initiation
- Have prediabetes
- Have type 2 diabetes
- Have health conditions that put the subject at risk to participate in exercise during this study
- Have atypical or Grade 2b diabetic sensorimotor polyneuropathy
- Have had more than one lifetime event of hospitalization for diabetic ketoacidosis

## 2.3 SAMPLE SIZE

We will include a total of **168 participants** in the current investigation – 84 participants with T1D, and 84 participants without T1D. Please see a breakdown of sample size by sex and age in the chart below. Groups are stratified in this way to ensure that appropriate comparisons between subjects with T1D and without T1D, overall AND stratified by young adult / older adult, as well as sex, may be made.

| Cohort | Age Group | Females | Males |
| --- | --- | --- | --- |
| T1D adults aged **18-30** (T1D duration <20 years) | Young adult | 18 | 18 |
| T1D adults aged **45-65** (T1D duration >30 years) | Older adult | 24 | 24 |
| Non-T1D adults aged **18-30** | Young adult | 18 | 18 |
| Non-T1D adults aged **45-65** | Older adult | 24 | 24 |

Power Calculation: Statistical analysis parameters were developed to allow sufficient power of Objective 1 primary outcome measures (the effect of T1D on skeletal muscle and microvascular health). Power calculations (2-sided test, power=0.8, alpha=0.05) were conducted using recently published and collected data from our lab. For Older adult subjects, mitochondrial density values (^26^; mu1 6.293, mu2 4.290, sigma 1.395) were used to reveal sufficient sample size for statistical power as n=17/sex/group. For Young adult subjects, Pax7-positive nuclei per 100-myofibers (indicative of skeletal muscle satellite cell content; preliminary data not shown herein; mu1 17.29, mu2 13.86, sigma 3.08) were used to reveal sufficient sample size for statistical power as n=13/sex/group. Both values were increased to a final sample size of older adult: n=24/sex/group and young adult: n=18/sex/group to provide a 40% excess to account for attrition and variability in initial aerobic fitness and responses to exercise training (exercise data was not available to derive appropriate power calculations). A full breakdown between groups can be seen in the table above. The longitudinal and paired nature of our methods further strengthens our statistical power for pre- and post- intervention analyses.

# 3. STUDY DESIGN AND MEASUREMENT INSTRUMENTS

## 3.1 STUDY SUMMARY

We will include a total of **168 participants** in the current investigation, as outlined above.

This study will require five visits, which can be seen in the study schematic timeline, below. All visits and data collection will be conducted within the Ivor Wynne Centre at McMaster University.

## 3.2 DETAILS OF INITIAL STUDY DOCUMENTATION

Prior to the baseline study visit, all subjects will complete the following surveys / documents / tests to confirm their eligibility and suitability to participate in this investigation:

1. Standard medical questionnaire
2. CSEP Get Active Questionnaire and CSEP Get Active Reference Document
3. Global Physical Activity Questionnaire (GPAQ)
4. Seven-day food log
5. Informed consent form
6. Tissue consent form

All information acquired from phone calls or emails sent to interested participants prior to completing the documents above will be kept strictly confidential. Participants will be encouraged to contact any of the investigators if they have any questions or concerns about the study. A copy of all signed consent forms will also be added to each participants data file.

## 3.3 DETAILS OF STUDY VISITS

Participants will not be permitted to consume alcohol or caffeine for 24 hours prior to each study visit. All participants will be instructed to abstain from participating in sports or conducting exercise of any kind for 24 hours prior to each visit. Exceptions may be made if the individual cycles to work, but this must be discussed with investigators.

Participants will undergo the following assessments. Study visits that include the methods detailed below are specified for each evaluation and can be seen on the study timeline and schematic above (Section 3.1 Study Summary).

**HEALTH QUESTIONNAIRE**

Participants will complete the following health questionnaires. Please find these questionnaires attached to the online interface of this ethics document submission.

- DASS-21 is a 21-item self-report scale designed to measure negative emotional states of depression, anxiety, and stress. The essential function of this established questionnaire is to assess severity of the core symptoms of depression, anxiety, and stress as part of the broader task of clinical assessment.^31–33^
- ONLY PARTICIPANTS WITH T1D: The DIDP (DAWN Impact of Diabetes Profile) is a six question, single-factor survey that has been shown to reliably measure the perceived impact of diabetes on quality of life.^34, 35, 44^

**BODY COMPOSITION AND SYSTEMIC EVALUATION**

- DEXA scan will be conducted to assess body composition, including fat, bone, and muscle mass. This procedure takes approximately seven minutes, during which the participant will lay still while a sensor passes over their body. Anthropometric measurements of height and weight will be recorded, and body mass will be calculated using the equation BMI = weight/(height)^2^. These metrics are required to perform the DEXA scan.
- Blood sampling: A small needle will be inserted into a forearm vein and a blood sample will be drawn (approximately 20 mL). After the blood sample has been taken, pressure will be placed on the site to minimize bleeding.

Blood is being sampled for analysis of primary measures (including glucose, insulin, free fatty acids, cholesterol, triglycerides, HDL, LDL, HbA1c, lactate) and secondary measures (including serum creatine kinase, creatinine, urea, uric acid, potassium, PAI-1, and tryptophan metabolites (kynurenines)).

**MACROVASCULAR MEASURES**

- Blood pressure will be measured in triplicate with at least one minute rest between measurements using an automated oscillometric device. These measurements will be conducted while the participant is seated and following a ten-minute resting period.
- Arterial stiffness will be measured using pulse wave velocity (PWV). The time it takes a pulse to travel between two superficial arterial regions of interest (carotid-femoral and femoral-foot) is accomplished using applanation tonometry and is an indicator of central and peripheral arterial stiffness. Two applanation tonometers (SPT-301, Millar Instruments) will be placed superficially on the outlined regions of interest for 20 heart cycles to calculate central and peripheral arterial stiffness using the formula: PWV = distance / transit time. This assessment will be performed at rest and 1-, 10-, and 20-minutes following the test of aerobic fitness.
- Carotid intima-media thickness (cIMT) is the measure of the thickness of the common carotid artery walls and can be indicative of arterial plaque burden. B mode ultrasound will be used to capture images of the common carotid artery which will later be analyzed using semi-automated edge tracking software (Arterial Measurement Systems, AMS).
- Carotid distensibility (CD) is a measure of carotid artery elasticity or the rigidity of the vessel wall. Increased or large compliance values correspond to decreased or diminished arterial stiffness. Arterial compliance reflects the ability of an artery to expand and recoil with cardiac pulsation and relaxation. Decreased central arterial compliance has been identified as an independent risk factor for future cardiovascular disease. This assessment involves the simultaneous measurement of B mode ultrasound (Vivid q, GE Medical Systems) of the right carotid artery and applanation tonometry (SPT-301, Millar Instruments) of the left carotid artery for 10 heart cycles.
- Endothelial-dependent vasodilatory function will be assessed using brachial artery flow mediated dilation (FMD).^30^ FMD represents the dilatory capacity of an artery in response to a rapid increase in shear stress. It involves the placement of a blood pressure cuff around the forearm distal to the elbow. The cuff will be inflated above systolic blood pressure to ~200 mmHg (to cease arm blood flow) and held at this pressure for five minutes. Continual measures of brachial artery diameter and blood flow velocity will be obtained using Doppler ultrasound (Vivid q, GE Medical Systems). A 12MHz probe will be placed on the upper arm (below the biceps) and moved until the best signal is found. Images of brachial artery diameter and blood flow velocity will be taken at rest (before cuff inflation), prior to cuff deflation (end of five-minute ischemic period) and following cuff deflation for three minutes. FMD will be calculated using this formula: FMD% = ((peak diameter - baseline diameter) / baseline diameter) × 100%. These measurements can be used to quantify endothelial-dependent function and are also approved under the HiREB ethics protocol #8000.

**STRENGTH AND AEROBIC FITNESS TESTING**

- Muscle strength testing: Quadricep maximal isometric strength (maximal voluntary contraction; MVC) will be measured using an isometric dynamometer (Biodex). The protocol will consist of three five-second periods during which participants will be encouraged to maximally push against the padded lever strapped to their leg, with 30 second breaks between each repetition. The lever will be set to a knee joint angle of 90 degrees. Hand grip strength will also be tested using a hand grip dynamometer connected to a commercially available data acquisition unit (Powerlab). Bilaterally, participants will perform two MVCs on the handgrip dynamometer, and if the two measurements differ by 5% on the same hand, a third will be performed.
- Aerobic fitness testing: Depending on the success of the CSEP Get Active Questionnaire, a PWC130 or VO_2_Peak stationary bike test (Lode, Excalibur Sport) will be conducted to assess whole body aerobic fitness and exercise capacity. For individuals that answer “Yes” to any questions of the CSEP Get Active Questionnaire, and successfully complete the CSEP reference document, a PWC130 test will be performed: participants will complete two consecutive six-minute rides on a stationary bike. The resistance during these rides will have been selected to produce a heart rate of approximately 100-110 beats per minute in the first ride, and approximately 130-140 beats per minute in the second ride. A tiered system (i.e., PWC140, PWC150) will be used if required. For individuals that answer “No” to all of the CSEP Get Active Questionnaire, a VO_2_Peak test will be performed: participants will complete a cycling program with an incremental increase in resistance until exhaustion. The warm-up period will consist of two minutes at 50 Watts, followed by an incremental increase of one Watt each two-seconds. Successful achievement of VO_2_Peak (ml/kg/min) is obtained, and testing will stop, when any of the following are met: participant heart rate reaches their predicted maximum, respiratory exchange ratio exceeds 1.1, or when volitional exhaustion is reached.

**INTRAMUSCULAR MEASURES**

- Motor neuron evaluation: Non-invasive high-density surface electromyography (EMG; Sessantaquattro 64-channel EMG) will be used to determine changes in the neural control of muscle force production. Namely, motor unit properties such as recruitment threshold, discharge rate, and conduction velocity will be evaluated to identify possible alterations in the neural strategies regulating force production.

To begin, the skin over the quadricep will be shaved and cleansed. EMG signals will be initially recorded during a brief voluntary contraction during which a non-adhesive dry electrode will be moved over the skin to detect the location of the innervation zone and confirm muscle fiber orientation/direction. Surface EMG signals of the vastus lateralis will then be recorded with an appropriately positioned two-dimensional adhesive electrode grid whose cavities will be filled with conductive paste once affixed to the skin. A strap electrode will be dampened with water and positioned on the medial malleolus of the right leg to be used as a ground electrode. The EMG signals will be recorded in a mono-polar mode and converted to digital data through a multi-channel amplifier for evaluation and analysis.

- Microvascular function and perfusion will be evaluated within the quadricep (vastus lateralis) using the Contrast Enhanced UltraSound (CEUS) method.

CEUS uses gas-filled lipid microspheres with thin permeable shells that are 1-3µm in size. Once the microspheres are released into systemic circulation (via intravenous infusion through a forearm vein), they act as acoustic reflectors to enhance the contrast in collected ultrasound images. The microspheres are smaller than red blood cells, which allows them passage through the microcirculation, but prevents them from diffusing through vessel walls. Their echogenic properties allow the quantification of the level of contrast within a tissue over time which indicates the overall perfusion capacity.^39^ This technique records the contrast-enhanced acoustic time-intensity curve at low mechanical index (MI) (MI = .8) during steady state intravenous microbubble infusion. Briefly, once steady state is achieved, a high MI “flash” (MI = 1.32) is implemented to destroy microbubbles in the region of interest, where microbubble replenishment curves are subsequently recorded using curve-fitting techniques. High MI “flashes” and subsequent low MI recordings will be performed for the assessment of microvascular blood flow and reactivity at rest and following unilateral isotonic knee extensions at 50% 1-RM, respectively.^48,49^ Sulfur hexafluoride-containing lipid microspheres (SonoVue; Bracco, Milan, Italy) are activated by a vial mixer (Vialmix, Lantheus) at 75Hz for 45 seconds, after which the activated microbubbles are prepared for intravenous infusion.^42^ Preparation of activated microbubbles is carried out by an experienced operator, strictly following the instructions provided by SonoVue. Infusion (2ml/min for one minute, then 1ml/min thereafter) and venous catheterization (antecubital vein) will be performed by a trained operator.

Microbubble appearance will be assessed within the quadricep muscles, and slope of bolus-muscle-perfusion, wash-in time, maximum acoustic intensity, and integral of peak intensity will be calculated from both legs to evaluate microvascular function. These measurements are also approved under the HiREB ethics protocol #8000.

- The resting muscle biopsy procedure takes approximately 20 minutes and involves the removal of a small piece of muscle. Dr. Irena Rebalka, supervised by Dr. Dinesh Kumbhare, will clean an area on the thigh and inject a small amount of local lidocaine anesthetic. She will then make a small incision (~4-5mm) in the skin into which the biopsy needle will be inserted. A small piece of muscle (~100 mg) will be removed by the biopsy needle. The incision will then be stitched closed and wrapped in a tensor bandage.

Muscle is being sampled for: mitochondrial bioenergetic measures, including carbohydrate and fat oxidation, as well as reactive oxygen species measures; histological and immunoblotting / fluorescent measures; electron microscopy; transcriptomic analysis on a select number of study participants.

**CONTINUOUS HEART RATE AND ACTIVITY MONITORING**

**Continuous throughout all 18 weeks of study**

- ALL participants will be provided with a Garmin Venu Sq watch following confirmation of eligibility to participate in this investigation. ALL participants will be instructed to wear this watch during the day and night for the entire duration of this study.
- On-the-wrist health and wellness monitoring will provide all participants with an overview of the following metrics, whose data will also be collected for the duration of this study:
  - Heart rate
  - Blood oxygen saturation during the day and during sleep
  - Physical activity intensity and duration
  - Hours and quality of sleep
  - Caloric expenditure
  - Respiration rate

**CONTINUOUS GLUCOSE MONITORING – ONLY PARTICIPANTS WITH T1D**

**Continuous throughout all 18 weeks of study**

- Individuals with T1D will also have their continuous glucose monitoring (CGM) device connected for time-in-target measures and evaluation for the entire duration of this study. If participants with T1D do not already use a CGM device, this device will be provided for their use for the entirety of their study participation.

## 3.4 DETAILS OF EXERCISE TRAINING

As detailed in the study timeline (section 3.1, above), individuals will participate in two blocks of exercise training – the first (exercise training in the study timeline) being twelve weeks in duration, and the second (exercise re-training in the study timeline) being four weeks in duration.

To model the current Diabetes Canada exercise guidelines^45^ as well as the current exercise recommendations of the Public Health Agency of Canada via the Canadian Society for Exercise Physiology,^46^ the following exercise guidelines will be provided to study participants.

- Partake in at least 150 minutes of moderate-to-vigorous-intensity aerobic exercise each week**.** Using your Garmin watch to monitor your heart rate during activity, activity minutes will be counted if they are WITHIN or ABOVE heart rate “Zone 3”, also called the “Aerobic” heart rate zone. You enter this zone when your heart rate (heart beats per minute) reaches 64% of your maximum calculated heart rate. Bouts of exercise will count toward your aerobic activity minutes if your heart rate moves into this “Aerobic” heart rate zone for a minimum of 10 consecutive minutes.
- Partake in resistance exercise two to three times per week. While this exercise does not require you to be in any specific heart rate zone, activity should continue for a minimum of ten consecutive minutes.
- Have no more than two consecutive rest days without exercise.

In addition to these guidelines, a suggested exercise calendar and home exercise video guide will be provided to participants to ensure ease of exercise participation and a consistent regime across all study subjects. Briefly, scheduled aerobic training (including high intensity interval training and brisk walks), and resistance training will be undertaken up to five days per week following an at-home exercise regime.

Participants will be educated in all exercises to ensure safety and competency. A lead-in phase, which will occur during weeks one and two of the exercise training period, replaces several structured exercise days with brisk walks. This will be utilized to introduce exercise and improve retention. The full exercise regime will commence from week three onwards. Participants with T1D will be directed to monitor their CGM to facilitate safety and educate them on glucose responses to exercise.

## 3.5 DETAILS OF DETRAINING PERIOD

Upon completion of the twelve-week exercise training period, all study participants will undergo a seven-day detraining period consisting of unilateral (single-leg) knee immobilization using a hinged knee-joint immobilization brace. While on the study participant, this brace will be locked at 60° of knee flexion. This position will ensure the foot is off the ground to prevent normal weight-bearing and will also allow knee extensors to stay relaxed.^47-50^ For ambulation, and to ensure no weight-bearing on the immobilized leg, participants will be provided with axillary crutches. Crutches will be adjusted according to the height of the participant, and training on the proper use of crutches, including navigation of stairs, doors, and other community obstacles, will be provided.

Participants will be instructed only to remove the knee brace in bed, prior to sleep. During bathing, participants will be instructed not to remove the brace, but to keep it dry by covering it with a large plastic bag provided by the research team. Participants will also be given a stocking to wear underneath the brace, which will be measured to extend from the proximal thigh to the ankle. The stocking is intended to mitigate discomfort and minimize the risk of skin irritation from the brace. Like the immobilization brace, this will be worn at all times and only removed during sleep.

In accordance with previous knee joint immobilization studies, participants will perform twice daily (morning and evening) range of motion movements of the ankle and knee to minimize the risk of vascular or muscular complications due to immobilization.^50-52^ Movements will be performed while lying in bed, and consist of knee flexion, ankle pumps, and leg lowers. A demonstration of these activities will be provided to participants prior to immobilization.

## 3.5 DETAILS OF RE-TRAINING PERIOD

As detailed in the study timeline (section 3.1, above), following completion of the detraining period, subjects will return to the exercise training plan for four weeks. Exercise training details are identical to those described in Section 3.4 – Details of Exercise Training, with the exception that a lead-in phase will not be used within the re-training period.

# 4. DATA ANALYSIS

Variables will be analyzed via a computerized statistical package (SPSS and GraphPad Prism). Differences in baseline measures between subjects with T1D and matched control participants, overall and stratified by young adults and older adults, as well as sex, will be assessed using t-test (if normal) or Mann-Whitney U test (otherwise) and chi-square or Fisher’s exact test for binary/categorical variables. Changes in baseline to post-training to post-detraining metrics for each group (male / female, young adult / older adult, T1D / non-T1D) will be examined graphically then using paired t-tests or Wilcoxon signed-rank tests, as appropriate. ANCOVA will be used to assess change score differences between groups and to assess sex differences using interaction terms. Despite subject matching, covariate analyses will be conducted to ensure that effects are adequately identified. P-values < 0.05 will be considered statistically significant. Additionally, due to its particular interest when studying disease populations, we will also calculate effect size using Hedges' g test to ensure that clinical relevance is considered along with traditional p-values.

# 5. BUDGET OVERVIEW

Funding source: Canadian Institutes of Health Research (CIHR) – Project 180340

**Total budget: $875,925 for five years**

**TRAINEES**

**GRADUATE STUDENTS:** $72,000

Funding for four MSc graduate students for a two-year period each. MSc students will be responsible for assisting with subject recruitment, data collection, and all analysis.

**POSTDOCTORAL FELLOW:** $300,000

The day-to-day conduct of studies outlined in this project requires a full-time dedicated team member. This postdoctoral fellow will be responsible for study management, conduct, and analysis of all variables for all five years.

**CONSUMABLES**

**CLINICAL AND LAB CONSUMABLES:** $175,000

$35,000 per year for five years based on historical supply use. Biopsies, blood collection: surgical disposables, lidocaine anesthetic, syringes, needles, vacutainer barrels, blood tubes. Vascular metric disposables: Ultrasound gel, electrodes, probe maintenance. Neuromuscular metric disposables: Probes, electrodes. Mitochondrial respiration reagents: buffer chemicals, inhibitors, metabolic substrates, fluorophores. Disposables: Pipette tips, tubes, gloves, kimwipes, etc. Histology, immunofluorescence supplies: reagents related to microscopy (slides, coverslips, cryoprotectant, blades, fixatives, antibodies, serums). Other reagents: enzymes, Western blotting reagents.

**EQUIPMENT AND FACILITIES**

**EQUIPMENT AND FACILITIES:** $205,240

Usage of all necessary equipment (high-density EMG, microvascular ultrasound, microscope), sample processing (blood to core processing facility, transcriptomic analysis on a subset of samples), and clinic usage (biopsy material autoclave).

**DEXCOM CONTINUOUS GLUCOSE MONITORS:** $22,000

To review time-in-target at baseline, after single bouts of exercise, and after the study training and detraining periods. This cost includes devices, consumables, and software.

**DEXA SCANS:** $33,600

Five scans per subject, and $40 per scan.

**GARMIN VENU SQ ACTIVITY AND HEART RATE MONITORS:** $26,085

Subjects will wear activity monitors throughout the duration of the study to record daily physical activity and heart rate, among other measures. Participants will keep these watches to acknowledge study participation following study completion.

**SUBJECT COMPENSATION**

**168 SUBJECTS:** $42,000

In addition to keeping the Garmin Venu Sq watch that was used for the duration of the study, all study participants will also be compensated $250 each for study participation and all travel compensation inclusively.

# 6. SUBJECT RECRUITMENT

## 6.1 OVERVIEW

Participants will be recruited in the following ways:

- **Local Advertisement:** Recruitment posters will be advertised across the McMaster campus, including the McMaster University Medical Centre, David Braley Athletic Centre, and the Ivor Wynne Centre. Posters will also be posted internally, and advertised in other HHS hospitals, as well as off-campus including cafes, fitness centers, and local stores.
- **Social Media:** Recruitment posters will be shared through active lab-specific and co-investigator Facebook, and Twitter accounts.
- **T1D Online Social Communities:** ConnectedinMotion and BeyondType1 as well as other T1D organizations with an online presence will share our recruitment poster and advertise our study online and on Twitter.
- **Classified Ads:** We will post classified ads in the following newspapers: Hamilton Spectator, Hamilton News, Niagara Falls Review, and Inside Halton. We will also post classified ads online on the Kijiji website.

## 6.2 RECRUITMENT POSTERS

Separate posters have been created to recruit young adult T1D participants, older adult T1D participants, young adult non-T1D participants, and older adult non-T1D participants. Please find these posters attached to the online interface of this ethics document submission.

## 6.3 SOCIAL MEDIA, ONLINE, CLASSIFIED AD RECRUITMENT

Through Dr. Thomas Hawke’ lab website (hawkelabca.com) and his active Twitter account, recruitment posters will be posted and tweeted, respectively. Online T1D communities will also be provided recruitment posters to share. Recruitment posters as well as text found on recruitment posters will be used for classified advertisement placements.

# 7. LITERATURE CITED

1. Iscoe KE, Campbell JE, Jamnik V, Perkins BA, Riddell MC. Efficacy of Continuous Real-Time Blood Glucose Monitoring During and After Prolonged High-Intensity Cycling Exercise: Spinning with a Continuous Glucose Monitoring System. *Diabetes Technol Ther*. 2006;8(6):627-635.

2. Purnell JQ, Zinman B, Brunzell JD, DCCT/EDIC Research Group. The Effect of Excess Weight Gain With Intensive Diabetes Mellitus Treatment on Cardiovascular Disease Risk Factors and Atherosclerosis in Type 1 Diabetes Mellitus. *Circulation*. 2013;127(2):180-187.

3. Rodrigues TC, Veyna AM, Haarhues MD, Kinney GL, Rewers M, Snell-Bergeon JK. Obesity and coronary artery calcium in diabetes: the Coronary Artery Calcification in Type 1 Diabetes (CACTI) study. *Diabetes Technol Ther*. 2011;13(10):991-996.

4. Nadeau KJ, Regensteiner JG, Bauer TA, et al. Insulin Resistance in Adolescents with Type 1 Diabetes and Its Relationship to Cardiovascular Function. *J Clin Endocrinol Metab*. 2010;95(2):513-521. doi:10.1210/jc.2009-1756

5. Højlund K, Beck-Nielsen H. Impaired Glycogen Synthase Activity and Mitochondrial Dysfunction in Skeletal Muscle: Markers or Mediators of Insulin Resistance in Type 2 Diabetes? *Curr Diabetes Rev*. 2006;2(4):375-395. doi:10.2174/1573399810602040375

6. Soedamah-Muthu SS, Fuller JH, Mulnier HE, Raleigh VS, Lawrenson RA, Colhoun HM. All-cause mortality rates in patients with type 1 diabetes mellitus compared with a non-diabetic population from the UK general practice research database, 1992–1999. *Diabetologia*. 2006;49(4):660-666. doi:10.1007/s00125-005-0120-4

7. Jensen J, Aslesen R, Ivy JL, Brors O. Role of glycogen concentration and epinephrine on glucose uptake in rat epitrochlearis muscle. *Am J Physiol Metab*. 1997;272(4):E649-E655. doi:10.1152/ajpendo.1997.272.4.E649

8. Shulman GI, Rothman DL, Jue T, Stein P, DeFronzo RA, Shulman RG. Quantitation of Muscle Glycogen Synthesis in Normal Subjects and Subjects with Non-Insulin-Dependent Diabetes by 13C Nuclear Magnetic Resonance Spectroscopy. *N Engl J Med*. 1990;322(4):223-228. doi:10.1056/NEJM199001253220403

9. The Diabetes Control and Complications Trial Research Group. The effect of intensive treatment of diabetes on the development and progression of long-term complications in insulin-dependent diabetes mellitus. *N Engl J Med*. 1993;329(14):977-986.

10. Schauer IE, Snell-Bergeon JK, Bergman BC, et al. Insulin Resistance, Defective Insulin-Mediated Fatty Acid Suppression, and Coronary Artery Calcification in Subjects With and Without Type 1 Diabetes. *Diabetes*. 2011;60(1):306-314. doi:10.2337/db10-0328

11. Yip J, Mattock MB, Morocutti A, Sethi M, Trevisan R, Viberti G. Insulin resistance in insulin-dependent diabetic patients with microalbuminuria. *Lancet (London, England)*. 1993;342(8876):883-887. doi:10.1016/0140-6736(93)91943-g

12. Orchard TJ, Chang Y-F, Ferrell RE, Petro N, Ellis DE. Nephropathy in type 1 diabetes: A manifestation of insulin resistance and multiple genetic susceptibilities? *Kidney Int*. 2002;62(3):963-970. doi:10.1046/j.1523-1755.2002.00507.x

13. Donga E, Dekkers OM, Corssmit EPM, Romijn JA. Insulin resistance in patients with type 1 diabetes assessed by glucose clamp studies: systematic review and meta-analysis. *Eur J Endocrinol*. 2015;173(1):101-109. doi:10.1530/EJE-14-0911

14. Cleland SJ, Fisher BM, Colhoun HM, Sattar N, Petrie JR. Insulin resistance in type 1 diabetes: what is “double diabetes” and what are the risks? *Diabetologia*. 2013;56(7):1462-1470.

15. Martin FI, Stocks AE. Insulin sensitivity and vascular disease in insulin-dependent diabetics. *BMJ*. 1968;2(5597):81-82. doi:10.1136/bmj.2.5597.81

16. DeFronzo RA, Hendler R, Simonson D. Insulin Resistance is a Prominent Feature of Insulin-dependent Diabetes. *Diabetes*. 1982;31(9):795-801. doi:10.2337/diab.31.9.795

17. Kilpatrick ES, Rigby AS, Atkin SL. Insulin Resistance, the Metabolic Syndrome, and Complication Risk in Type 1 Diabetes: Double diabetes in the Diabetes Control and Complications Trial. *Diabetes Care*. 2007;30(3):707-712. doi:10.2337/dc06-1982

18. Public Health Agency of Canada. *Highlights: Diabetes in Canada: Facts and Figures from a Public Health Perspective*.; 2011.

19. Jakicic JM, Kraus WE, Powell KE, et al. Association between Bout Duration of Physical Activity and Health: Systematic Review. *Med Sci Sports Exerc*. 2019;51(6):1213-1219.

20. J DP-C, A G-H, RM A-R, et al. Replacing Sedentary Time: Meta-analysis of Objective-Assessment Studies. *Am J Prev Med*. 2018;55(3):395-402.

21. Pedersen BK, Febbraio MA. Muscles, exercise and obesity: skeletal muscle as a secretory organ. *Nat Rev Endocrinol*. 2012;8(8):457-465. doi:10.1038/nrendo.2012.49

22. Egan B, Zierath JR. Exercise Metabolism and the Molecular Regulation of Skeletal Muscle Adaptation. *Cell Metab*. 2013;17(2):162-184. doi:10.1016/j.cmet.2012.12.012

23. Hawley JA, Hargreaves M, Joyner MJ, Zierath JR. Integrative Biology of Exercise. *Cell*. 2014;159(4):738-749. doi:10.1016/j.cell.2014.10.029

24. Cartee GD, Hepple RT, Bamman MM, Zierath JR. Exercise Promotes Healthy Aging of Skeletal Muscle. *Cell Metab*. 2016;23(6):1034-1047. doi:10.1016/j.cmet.2016.05.007

25. Röckl KSC, Witczak CA, Goodyear LJ. Signaling mechanisms in skeletal muscle: Acute responses and chronic adaptations to exercise. *IUBMB Life*. 2008;60(3):145-153.

26. Monaco CMF, Tarnopolsky MA, Dial AG, et al. Normal to enhanced intrinsic mitochondrial respiration in skeletal muscle of middle- to older-aged women and men with uncomplicated type 1 diabetes. *Diabetologia*. Published online August 14, 2021:1-17.

27. Dial AG, Monaco CMF, Grafham GK, Patel TP, Tarnopolsky MA, Hawke TJ. Impaired Function and Altered Morphology in the Skeletal Muscles of Adult Men and Women With Type 1 Diabetes. *J Clin Endocrinol Metab*. 2021;106(8):2405-2422.

28. Monaco CMF, Bellissimo CA, Hughes MC, et al. Sexual dimorphism in human skeletal muscle mitochondrial bioenergetics in response to type 1 diabetes. *Am J Physiol Metab*. 2020;318(1):E44-E51. doi:10.1152/ajpendo.00411.2019

29. Monaco CMF, Hughes MC, Ramos S V., et al. Altered mitochondrial bioenergetics and ultrastructure in the skeletal muscle of young adults with type 1 diabetes. *Diabetologia*. 2018;61(6):1411-1423. doi:10.1007/s00125-018-4602-6

30. Thijssen DHJ, Black MA, Pyke KE, et al. Assessment of flow-mediated dilation in humans: a methodological and physiological guideline. *Am J Physiol Circ Physiol*. 2011;300(1):H2-H12. doi:10.1152/ajpheart.00471.2010

31. Gomez R, Summers M, Summers A, Wolf A, Summers J. Depression Anxiety Stress Scales-21. *http://dx.doi.org/101177/1073191113514106*. 2014;21(4):418-426.

32. Mizzi AL, McKinnon MC, Becker S. The Impact of Aerobic Exercise on Mood Symptoms in Trauma-Exposed Young Adults: A Pilot Study. *Front Behav Neurosci*. 2022;16.

33. Bener A, Al-Hamaq O, Dafeeah E. High Prevalence of Depression, Anxiety and Stress Symptoms Among Diabetes Mellitus Patients. *Open Psychiatr J*. 2011;5(1):5-12. doi:10.2174/1874354401105010005

34. Holmes-Truscott E, Cooke DD, Hendrieckx C, Coates EJ, Heller SR, Speight J. A comparison of the acceptability and psychometric properties of scales assessing the impact of type 1 diabetes on quality of life—Results of ‘YourSAY: Quality of Life.’ *Diabet Med*. 2021;38(6):e14524. doi:10.1111/DME.14524

35. Holmes-Truscott E, Skovlund SE, Hendrieckx C, Pouwer F, Peyrot M, Speight J. Assessing the perceived impact of diabetes on quality of life: Psychometric validation of the DAWN2 Impact of Diabetes Profile in the second Diabetes MILES – Australia (MILES-2) survey. *Diabetes Res Clin Pract*. 2019;150:253-263. doi:10.1016/J.DIABRES.2019.03.020

36. Celermajer DS, Sorensen KE, Gooch VM, Spiegelhalter DJ, Miller OI, Sullivan ID, Lloyd JK, & Deanfield JE. Non-invasive detection of endothelial dysfunction in children and adults at risk of atherosclerosis. *Lancet*, 1992; 340: 1111-15.

37. Bellamkonda K, Williams M, Handa A, & Lee R. Flow Mediated Dilatation as a Biomarker in Vascular Surgery Research. *J Atheroscler Thromb*. 2017; 24(8):779-787.

38. Shenouda N, Preist SE, Rizzuto VI, MacDonald MJ. Brachial artery endothelial function is stable across a menstrual and oral contraceptive pill cycle but lower in premenopausal women than in age-matched men. *Am J Physiol Heart Circ Physiol.* 2018; 315: H366-H374.

39. Chen SY, Wang YW, Chen WS, & Hsiao MY. Update of Contrast  enhanced Ultrasound in Musculoskeletal Medicine: Clinical Perspectives – A Review. *Journal of Medical Ultrasound*, 2023. 31(2): 92-100.

40. Mitchell WK, Phillips BE, Williams JP, Rankin D, Smith K, Lund JN, Atherton PJ. Development of a new Sonovue contrast-enhanced approach reveals temporal and age-related features of muscle microvascular responses to feeding. Physiol Rep, 2013: 1(5): e00119.41. Sjoberg KA, Rattigan S, Hiscock N, Richter EA, Kiens B. A new method to study changes in microvascular blood volume in muscle and adipose tissue: real-time imaging in humans and rat. *Am J Physiol Circ Physiol*, 2011. 301:H450-8.

42. Herrod PJJ. Atherton PJ, Smith K, Williams JP, Lund JN, & Phillips BE. 6-weeks of high-intensity interval training enhances contractile activity induced vascular reactivity and skeletal muscle perfusion in older adults. *GeroScience*, 2021. 43: 2667-78.

43. Dunford EC, Au JS, Devries MC, Phillips SM, and MacDonald MJ. Cardiovascular aging and the microcirculation of skeletal muscle: using contrast-enhanced ultrasound. *Am J Physiol Circ Physiol,* 2018. 315(5): H1194-99.

44. Peyrot M, Burns KK, Davies M, et al. Diabetes Attitudes Wishes and Needs 2 (DAWN2): a multinational, multi-stakeholder study of psychosocial issues in diabetes and person-centred diabetes care. *Diabetes Res Clin Pract*. 2013;99(2):174-184.

45. Sigal R, Armstrong M, Bacon S, et al. Diabetes Canada 2018 Clinical Practice Guidelines for the Prevention and Management of Diabetes in Canada. *Can J Diabetes*. 2018;42(Suppl 1):S1-S325.

46. The Canadian Society for Exercise Physiology (CSEP). Canadian 24-Hour Movement Guidelines for Adults Aged 18-64 Years: An Integration of Physical Activity, Sedentary Behaviour, and Sleep. Published 2021. Accessed January 10, 2023. https://csepguidelines.ca/

47. Oates BR, Glover EI, West DW, Fry JL, Tarnopolsky MA, Phillips SM. Low-volume resistance exercise attenuates the decline in strength and muscle mass associated with immobilization. *Muscle Nerve*. 2010;42(4):539-546. doi:10.1002/MUS.21721

48. Inns TB, Bass JJ, Hardy EJO, et al. Motor unit dysregulation following 15 days of unilateral lower limb immobilisation. *J Physiol J Physiol*. 2022;600:4753-4769. doi:10.1113/JP283800

49. Abadi A, Glover EI, Isfort RJ, et al. Limb Immobilization Induces a Coordinate Down-Regulation of Mitochondrial and Other Metabolic Pathways in Men and Women. Published online 2009. doi:10.1371/journal.pone.0006518

50. Yasuda N, Glover EI, Phillips SM, Isfort RJ, Tarnopolsky MA. Sex-based differences in skeletal muscle function and morphology with short-term limb immobilization. *J Appl Physiol*. 2005;99(3):1085-1092.

51. Deschenes MR, Holdren AN, Mccoy RW. Adaptations to short-term muscle unloading in young and aged men. *Med Sci Sports Exerc*. 2008;40(5):856-863. doi:10.1249/MSS.0B013E318164F4B6

52. MacLennan RJ, Ogilvie D, McDorman J, et al. The time course of neuromuscular impairment during short-term disuse in young women. *Physiol Rep*. 2021;9(1):e14677. doi:10.14814/PHY2.14677

53. MacLennan RJ, Sahebi M, Becker N, Davis E, Garcia JM, Stock MS. Declines in skeletal muscle quality vs. size following two weeks of knee joint immobilization. *PeerJ*. 2020;2020(1):e8224. doi:10.7717/PEERJ.8224/SUPP-2
